# Guy's Hospital

**Published:** 1893-07-22

**Authors:** 


### THE INSTITUTIONAL WORKSHOP.

### WITHIN THE HOSPITALS.

### GUY'S HOSPITAL.

OPENING OF NEW LABORATORIES AND DENTAL SCHOOL BUILDINGS.

The growing popularity of the great school of medicine attached to Guy's Hospital is placed beyond dispute by the fact that the number of students (106) who entered for the full curriculum during the last academical year is the highest entry of which any record has been preserved. This remarkable popularity caused the total entry of new students to increase to 191—including in this numeration those who joined for short periods of study or special classes—a number which exceeds by thirty-six the total entry for the year of any other London medical school. Such figures are worthy of close attention, because this increase in the entry of students occurs in the first year when the new regulations of the General Medical Council have come into force, whereby a fifth year has been added to the professional curriculum. Monday, the 17th inst., was altogether a great day for Guy's Hospital and its medical school. In the morning a luncheon was given in the court room of the hospital, which is one of the most striking and interesting of the great halls attached to ancient buildings within the City boundaries. We wonder what the founder, Thomas Guy, thought of it all, as he looked on in silence out of the gold frame which encloses his portrait at the top of the hall! Had he been able to take part in the subsequent proceedings he would have seen the court on one side of the quadrangle roofed in as a tent and crammed to overflowing with medical men, professors and students, their friends and relations, who thronged in surprisingly large numbers in order to show by their presence the deep interest which they take in everything that concerns the wellbeing and progress of this great medical school. Old Guys, that is to say, those portions which form the quadrangle, have undergone many alterations since the foundation of the hospital. Excellent and necessary as these improvements have been, the new buildings behind them exhibit features still more remarkable, as marking the advance and progress of medical science and teaching in this country to-day. There are, for instance, the college buildings, controlled by a warden, where the students can reside under conditions most favourable to study, and where those who are non-resident can obtain excellent food and necessary recreation without leaving the hospital grounds. The capacity and excellence of these arrangements were shown on Monday by the circumstance that the luncheon given by the governors to the staff and their guests was supplied from the college kitchen. This is a small matter in itself, but it emphasizes the development and progress which has gone on in every department so far as Guy's Hospital Medical School is concerned.

THE LUNCHEON.

Mr. Henry Hucks Gibbs, the president, presided, and was supported by the Right Honourable Sir John Lubbock, Bart., M.P., Sir Henry Peek, Sir George Young, Sir Trevor Lawrence, Sir W. Cameron Gull, Sir Edwin Saunders, Sir John Tomes, Sir Joseph Fayrer, Sir George Buchanan, Sir C. Freemantle, Professors Michael Foster and Odling, many of the consulting and acting honorary medical and surgical staff, representative governors and other friends and supporters of Guy's. As the luncheon progressed it was impossible to look round the tables, and to recognise one by one the great physicians and surgeons who have made Guy's what it is to-day without being struck by the fact that of late years honours have been somewhat erratically distributed among members of the medical profession, and that for some cause not quite apparent, the surgical branch of the profession has been practically forgotten. How is it, for example, that a man of the reputation and character of Mr. Thomas Bryant, who has so ably fulfilled the duties of President of the Royal College of Surgeons for some years, and Mr. Jonathan Hutchison, F.R.C.S., than whom no member of the profession has done more for the advancement of the surgery of his day and generation, should be still without any mark of her Majesty's favour? How is it, too, that so old, so able, and so famous a man as Dr. Wilks should have been passed by when others have been decorated? It has been truly said of Dr. Wilks that he is more trusted by the individual members of the medical profession when they are themselves ill than any other physician, and that he is, indeed, the general practitioner of the whole medical profession. We hope that the day is not far distant when due recognition will be paid to his merits, and so it may be made impossible to write of him—though such an epitaph would be a high honour and testimony to his worth—"Had he been less of a friend to the members of his profession in his day and generation; had he given more time to his own interests than he so nobly devoted to those of science and medicine, the world and the profession would have been the poorer, but Samuel Wilks would have individually benefited to an extent more commensurate with his merits." It was interesting to see sitting side by side the senior demonstrator of to-day and the demonstrator of forty years ago; to hear them discuss the past and present difficulties, and to find how much they were in accord as to the lines upon which anatomy must be taught, if a great school like Guy's Hospital is to maintain its position in the first rank.

After food, speech. This luncheon illustrated the disadvantages which public men of repute are often under, because they have apparently no friend to tell them that their remarks are pitched in so low a key that it is impossible for an audience to understand the drift, much less to follow intelligently the thread of their speeches. Sir John Lubbock spoke with exceptional ability, but his remarks—small as the hall was —were inaudible to the majority present. We have noticed the same thing when he has been speaking in the House of Commons, and were immensely struck with the drawback under which he labours in this respect at a small meeting in one of the smaller rooms in the Mansion House a few weeks ago when he was quite inaudible. So far as we could gather Sir John

Lubbock emphasised the sympathy he felt for Guy's, as M.P. for London University, because of the splendid work Guy's men have accomplished at its examinations. He congratulated teachers and students upon the wisdom which the Governors had displayed in keeping abreast of the times by affording the maximum of facility for acquiring knowledge at the Guy's medical school. He joked Sir Edwin Saunders and Sir John Tomes on the terrible array of instruments and dental chairs which adorned the new buildings, and expressed his indebtedness to the dental profession for the energy derived from a sound digestion and abundant rest, due to the powers of mastication supplied by the painless dentistry of modern times. Professor Odling and Sir Edwin Saunders followed, the former with a lengthy biographical sketch, which brought out the interesting fact that in old times the dissecting-room at Guy's was converted into a theatre for practical chemistry during the summer session. After the luncheon the company adjourned to the tent, where the prizes were distributed by Sir John Lubbock. A list of medalists and prizemen will be found in the "Student of Medicine" section of our present issue.

The New Buildings.

A visit to the new laboratories at Guy's Hospital comes as a revelation to the medical practitioner of 30 years' standing. If he visits the chemistry laboratories and sees all the arrangements for the practical teaching of this science, which includes, in fact, a small laboratory with ample appliances and chemicals for each member of a class of 60 students or upwards, he cannot fail to rub his eyes, and remember what practical chemistry was when he was a student. We have never seen anything so complete or thoroughly well done as these laboratories undoubtedly are. Any student who fails to pass a fair examination after a course in these buildings ought to be rusticated. It is true, no doubt, that the modern student has much more to learn than we had in our day, but the means for acquiring knowledge have so developed and are so complete that the dullest intellect must find it comparatively easy to progressively pass each of his examinations without the expenditure of an unreasonable amount of energy or brain power. In the same way the dental department is remarkable. It is wonderful to enter the dental laboratory, and to see similar provision for a much larger class of dental students, where practical work can be done under conditions which enable the teacher to exercise complete supervision over every student, and to compel him to master the work with which he is entrusted, or to stand convicted of idleness and incapacity. Upstairs, at the top of the buildings, that is of course as near heaven as possible, the medical school have placed a "chamber of consolation." This "chamber of consolation" contains so large a number of dental chairs, that we failed to count them successfully. Each chair is provided with the ordinary appliances and instruments to be met with in the consulting-room of a modern dentist, and here patients and students laboriously linger until each is satisfied with the work of the other. At least, in no other way can we account for the title which has been given to this chamber of horrors, for it is nothing else when seen in cold blood, in the absence of both the students and their (?) patients.

We heartily congratulate everybody connected with Guy's Hospital upon the opening of these well-planned buildings. We would advise all parents who have sons to place out in life to communicate with the Dean, and to take an early opportunity of inspecting them, so that they may realise what a splendid chance of success is placed within the reach of every young fellow who has the good fortune to become a student of Guy's Hospital in the present day. The wisdom of this advice will be driven home to each parent's mind who realizes that 25 per cent. of the dental students of Guy's Hospital are studying to become qualified medical practitioners as well as dental surgeons.

